# Cross-Cultural Validation of the Short Version of the Questionnaire of Olfactory Disorders—Negative Statements into Italian: Towards Personalized Patient Care

**DOI:** 10.3390/jpm12122010

**Published:** 2022-12-04

**Authors:** Francesca Pirola, Francesco Giombi, Fabio Ferreli, Andrea Costantino, Giuseppe Mercante, Giovanni Paoletti, Enrico Heffler, Giorgio Walter Canonica, Stefano Settimi, Eugenio De Corso, Giuseppe Spriano, Luca Malvezzi

**Affiliations:** 1 Otorhinolaryngology Head & Neck Surgery Unit, IRCCS Humanitas Research Hospital, Via Manzoni 56, Rozzano, 20089 Milan, Italy; 2 Department of Biomedical Sciences, Humanitas University, Via Rita Levi Montalcini 4, Pieve Emanuele, 20090 Milan, Italy; 3 Personalized Medicine, Asthma and Allergy, IRCCS Humanitas Research Hospital, Via Manzoni 56, Rozzano, 20089 Milan, Italy; 4 Department of Head and Neck and Sensory Organs, Catholic University of the Sacred Hearth, 00168 Rome, Italy; 5 Unit of Otorhinolaryngology-Head and Neck Surgery, A. Gemelli Hospital Foundation IRCCS, 00168 Rome, Italy

**Keywords:** questionnaire, olfactory dysfunction, quality of life, chronic rhinosinusitis, septal deviation

## Abstract

Given the high burden of olfactory dysfunction worldwide, recently increased due to the COVID-19 pandemic, it is mandatory to adopt a specific questionnaire to assess the impact of olfactory impairment on quality of life, to be used in clinical practice. The aim of this study is to adapt and validate the short version of the Questionnaire of Olfactory Disorders-Negative Statements (svQOD-NS) for Italian. In the pilot phase, the Italian version of the questionnaire (ITA-svQOD-NS) was produced following recommended guidelines. It was then given to 50 healthy subjects and 50 patients (affected by either nasal polyposis or septal deviation), and results were compared to those of other widely used questionnaires. Test-retest reliability was assessed on a sample of 25 patients. All 50 patients repeated the questionnaires at one and nine months after surgery. The internal consistency of ITA-svQOD-NS measured with Cronbach α was excellent (α = 0.92). The intraclass correlation coefficient for test-retest reliability was also optimal (0.93; 95%CI: 0.90–0.96). Concurrent validity tested with the Pearson coefficient was significant with all other tests administered; also, concerning responsiveness, statistically significant differences were obtained between pre- and post-operative conditions. ITA-svQOD-NS showed high internal consistency, test-retest reliability, and significant correlation with all most-used clinical questionnaires; thus, it can be efficiently applied to assess olfaction-related QoL in the Italian population.

## 1. Introduction

Olfactory dysfunction (OD) is a common clinical condition in otolaryngological clinical practice. It has been reported to affect more than 200,000 patients per year in the United States, with age-related incidence, especially after 60 years [1,2]. OD may be associated with several disorders, either otolaryngological (e.g., common viral upper airways affections, acute or chronic rhinosinusitis, and benign or malignant sinonasal tumors) [3], or neurological (e.g., Parkinson’s or Alzheimer’s disease) [4,5]. Recently, OD has been recognized as an alarming symptom that should raise suspicion for COVID-19 infection [6], especially in otherwise asymptomatic carriers [7,8]; it also proved to be useful in monitoring COVID-19 clinical evolution [9].

Despite its broad diffusion, the spectrum of quality of life (QoL) impairment due to olfactory disorders is often underrated [10]. In addition to obvious issues for workers whose job relates to olfactory performance, odor sensing is essential in a variety of circumstances, such as the ability to notice potential health risks (e.g., a gas leak, rotten food, etc.) or to enjoy recreational moments (e.g., meals, wine-tasting and scents, etc.). It has also been demonstrated that the impact of OD on QoL can eventually have psycho-emotional consequences that might lead to anxiety, isolation, and depression [11]. Consequently, for an optimal management of this clinical condition, a multidisciplinary approach should be considered [12,13,14].

In the diagnostic process, commonly used tools to assess OD include psychophysical olfactory tests such as the “Sniffin’ Sticks” (Burghart^®^), which uses pen-like odor-dispensing devices to test odor threshold, discrimination and identification, or the UPSIT test (Sensonics International^®^), made of a scratch-and-sniff booklet that is very suitable for self-administration by patients. In this context, disease-specific QoL questionnaires are not regularly adopted in common clinical practice. Other questionnaires that were developed to assess nasal conditions, such as the well-known 22-item Sinonasal Outcome Test (SNOT-22) [15] and the Visual Analogue Scale (VAS) for Olfaction [16], consider a broader set of symptoms without specifically focusing on OD and its impact on QoL. 

The Questionnaire of Olfactory Disorders-Negative Statements (QOD-NS) is a disease-specific questionnaire that was initially developed and proposed in 2005 by Frasnelli and Hummel, demonstrating good correlation with OD and good psychometric validity [17]. A short version of the QOD-NS (svQOD-NS) was published in 2019 by Mattos JL et al. using only seven selected questions from the original seventeen, achieving excellent correlations with both total and domain-specific scores [18]. In the era of personalized medicine, assessing the impact on QoL of olfactory-related symptoms could be useful in several clinical scenarios; however, the svQOD-NS is currently available only in English, Spanish and French. The aim of this study is to produce a cross-cultural validation of the svQOD-NS to allow its use in the Italian population.

## 2. Materials and Methods

**Figure 1:**
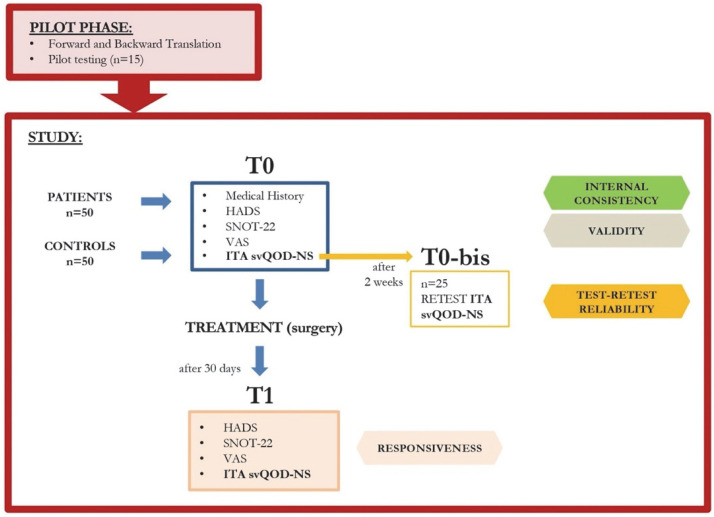
Design of the study.

**Figure 2:**
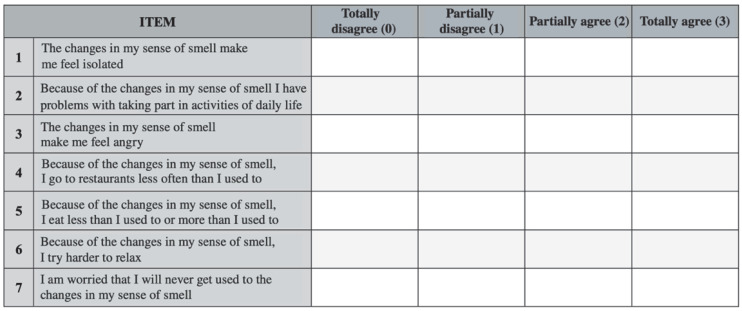
English version of Questionnaire of Olfactory Disorders—Negative Statements (svQOD-NS).

The study was performed in accordance with the ethical standards of the Declaration of Helsinki and its later amendments, and it was approved by the Ethical Committee of our Institute (IRCCS-ICH-IEC/3114). All patients signed informed consent to participate. The methodology of the study is shown in Figure 1. International guidelines [19] were followed to translate, review, and validate the original English version of the svQOD-NS for Italian (Figure 2). Two different initial Italian translations of the svQOD-NS were carried out by two independent otolaryngologists, both Italian and English mother-tongue speakers. The translated versions were sent to an expert panel of three Italian otolaryngologists to obtain a pooled version, which was backward-translated into English afterwards by an independent bilingual English-Italian speaker and compared to the original questionnaire. The language quality was reviewed. Eventually, a consensus on a pre-final version was achieved, and a pilot test for the comprehensibility of the questions was conducted, submitting the questionnaire to 15 Italian-speaking subjects.

**Figure 3:**
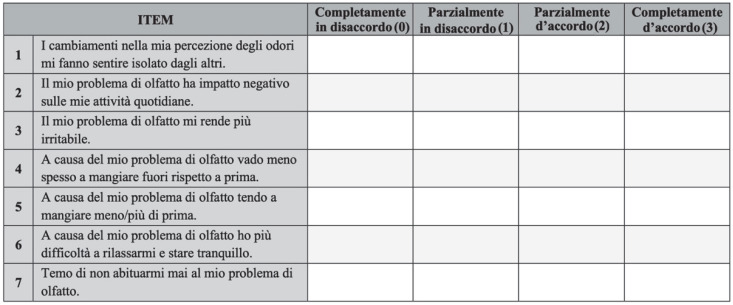
Italian version of Questionnaire of Olfactory Disorders—Negative Statements (ITA svQOD-NS).

Finally, in consideration of the adjustments derived from the pilot test, the final Italian version (ITA svQOD-NS) was formulated by the panel (Figure 3). For the validation of the ITA svQOD-NS, we gave the questionnaire to 100 adult subjects (50 cases and 50 controls), who subsequently accessed the Otolaryngology Outpatient Clinic of IRCCS Humanitas Research Hospital (Milan, Italy) between August and September 2021. Power analysis was carried out to confirm the appropriateness of the sample size for statistical significance.

Inclusion criteria for the case group were as follows: (1) age ≥ 18 years, and ability to read and understand Italian; (2) being affected by either septal deviation (SD) or chronic rhinosinusitis with nasal polyps (CRSwNP); (3) complaining of olfactory impairment (VAS for Olfaction arbitrarily set ≥4); (4) willing to give informed consent for the study; (5) having surgery for SD or CRSwNP planned by the end of September 2021. The diagnosis of SD or CRSwNP was carried at the patients’ referral, at the endoscopic examination routinely performed in the outpatient clinic. Patients in the control group were enrolled with a 1:1 ratio to cases, until we reached the set sample size. Inclusion criteria for the control group were as follows: (1) not being affected by any sinonasal disease, (2) age ≥ 18 years and ability to read and understand Italian, (3) not complaining of olfactory impairment to any extent; (4) willing to give informed consent. All control subjects tested negative for sinonasal disease or structural abnormality of the nose at endoscopic examination prior to enrollment. Any patient who revealed cognitive impairment (age- or disease-related, or under medication) in the Quick Mild Cognitive Impairment test (QMCI-I) [20] was excluded from the study, as well as those with a diagnosis of neuropsychiatric disorder.

After collecting patients’ demographic characteristics and clinical history, each subject answered disease-specific QoL questionnaires at different times during the study (Figure 1): (1) the 22-item Sinonasal Outcome Test (SNOT-22) [15], (2) VAS for Olfaction [16], (3) the Italian short version of the Questionnaire of Olfactory Disorders-Negative Statements (ITA svQOD-NS), and (4) the Hospital Anxiety and Depression Scale (HADS). The latter is a useful scale for general assessment of the severity of anxiety and depression in both primary care patients and the general population [21]. To measure test-retest reliability of the questionnaire, a subset of patients from the case group repeated the ITA svQOD-NS two weeks after the first administration, without taking any medication or treatment that would change their condition in the meantime. Then, all patients (case group) underwent surgery by the same surgical team, and the aforementioned tests (SNOT-22, VAS-olfaction, ITA svQOD-NS, and HADS) were re-administered at 1 and 9 months after the procedure, in order to assess questionnaire responsiveness.

Statistical analysis was conducted using IBM**^®^** SPSS Software for Macintosh, Version 26.0. The sample size was calculated with α-error set at 0.05 and β-error at 0.20, for a study power of 80%. Internal consistency, test-retest reliability, validity, and responsiveness of the ITA svQOD-NS were analyzed. Correlations between ITA svQOD-NS and the other questionnaires were measured with a Pearson coefficient. A one-way ANOVA test was used for comparing means, to assess differences between mean scores in the subgroups.

## 3. Results

One hundred adult subjects (50 cases and 50 controls) were enrolled. Patients’ characteristics are described in Table 1.

The minimum sample size to assess the responsiveness of the ITA svQOD-NS at one and nine months after surgery was 28 and 20, respectively. Considering cases and controls, the minimum sample size was 16 and was thus satisfied by our study population. A subset of 25 subjects repeated the ITA svQOD-NS after two weeks for test-retest reliability. All patients in the case group completed the ITA svQOD-NS, SNOT-22, VAS, and HADS before surgery, and repeated the same questionnaires at one and nine months after surgery. Results are shown in Table 2.

The internal consistency of the ITA svQOD-NS calculated with Cronbach α was 0.92, and it was still over 0.90 after single-item exclusion. The correlation of each item with all others combined (item-to-total correlation) was higher for item #3 and #6 (Table 3).

The intraclass correlation coefficient for test-retest reliability was 0.93 (95%CI: 0.90–0.96). Concurrent validity was tested by a Pearson coefficient (ρ) between ITA svQOD-NS and all other tests administered before surgery. Statistically significant results were obtained in all cases and correlation was higher with SNOT-22 (ITA svQOD-NS and SNOT-22: ρ = 0.61, *p <* 0.001; ITA svQOD-NS and VAS: ρ = 0.42, *p* = 0.01; ITA svQOD-NS and HADS: ρ = 0.42, *p* = 0.01). Subgroup analysis revealed that mean scores for each item of the ITA svQOD-NS and the total scores were significantly higher in patients who suffered from CRSwNP than in patients with septal deviation and healthy controls (Table 4 and Table 5; *p* < 0.001). With regard to responsiveness, significant differences were obtained by comparing means of each questionnaire collected pre- and post-operatively (Table 6).

## 4. Discussion

Given the high burden of OD worldwide, with its further increase during the COVID-19 pandemic [22], the cross-cultural validation of a questionnaire developed to assess OD-related QoL is needed for a standardized evaluation of the patients’ status. This also finds greater relevance in the context of the personalized medicine approach adopted in patients with nasal disorders [23]. As mentioned, across the literature, other questionnaires can be found which address nasal conditions and olfaction-related symptoms, such as the SNOT-22 [15] and VAS for Olfaction [16]. However, these instruments do not focus specifically on OD and to what extent it impacts patients’ QoL. The Questionnaire of Olfactory Disorders-Negative Statements (QOD-NS) is a disease-specific questionnaire that sheds light on this relevant yet underestimated issue. Psychometric validity of the questionnaire and good correlation with OD was demonstrated in the original publication by J. Frasnelli and T. Hummel; however, one of its shortcomings was the large number of questions it was made of, which could make it cumbersome and time-consuming to administer in outpatient settings [17]. Thus, in 2019, Mattos et al. published a shorter version of the QOD-NS (svQOD-NS) that could be more suitable to scenarios of everyday clinical practice [24]. Later, the same short version was validated by Chiesa-Estomba et al. in Spanish in 2020 [25] in a group of 40 patients affected by COVID19-related OD, extending the use of the svQOD-NS to a larger set of Spanish-speaking patients worldwide. Recently, the svQOD-NS has also been demonstrated to be a reliable tool in the French population, showing good internal consistency, test–retest reliability, as well as acceptable external and internal validity [26].

To date, there is no validated version of the svQOD-NS in Italian. In our study, we gave the translated ITA svQOD-NS to 50 patients and 50 control subjects, the former being represented by 25 subjects with nasal septum deviation (SD) only and 25 with diagnosis of CRSwNP, for whom surgery in our department was planned a short time later. Olfactory dysfunction (hyposmia/anosmia) is a typical symptom of CRSwNP, and its relevance in affecting QoL is largely reported across literature [27,28,29]. Also, despite being less common, studies have reported that patients affected by SD may experience olfactory dysfunction, with evidence of improvement after septoplasty [30,31,32]. For these reasons, we chose to include both patients with CRSwNP and SD to test the applicability of the ITA svQOD-NS in two categories of patients who reported OD due to different etiologies. All cases enrolled underwent surgery by the same surgeons and received the same postoperative treatments (within their diagnostic category), as well as follow-up examinations. They were asked to fill out the same questionnaires (SNOT-22, VAS-olfaction, ITA svQOD-NS, and HADS) at one and nine months after surgery, to test responsiveness. In this survey, we decided not to perform further olfactory testing, since it is demonstrated that objective olfactory thresholds do not correlate with the subjective perception of smell and may have a different impact on QoL in different subjects, which our questionnaire aims to explore [33].

The internal consistency of the ITA svQOD-NS, which expresses the homogeneity of the items and their consistency in measuring the real outcomes of the test, was excellent (Cronbach α ≥ 0.90). Test-retest reliability was likewise excellent (intraclass correlation coefficient ≥ 0.90); it quantified the consistency of the questionnaire results when repeated by the same patients two weeks after the first time, and without any change in the underlying condition, as no treatments were given to them. Concerning concurrent validity, which measures how closely a new test associates with well-established ones, the ITA svQOD-NS was compared to the SNOT-22, VAS-Olfaction and HADS both pre- and postoperatively, showing good accuracy results. The responsiveness of the questionnaire measured at one and nine months after surgery was good, also reflecting the expected efficacy of surgery in reducing symptoms of OD over a long follow-up period (Table 6). Finally, a subgroup analysis was performed to verify if results differed between patients affected by CRSwNP and those with SD (Table 5), showing statistically significant differences. It may be inferred, accordingly, that the questionnaire mirrored the difference in olfaction-related QoL that is perceived by CRSwNP patients (that is, greater; mean = 12.23 ± 5.31) and by those with only SD (that is, lower; mean = 4.53 ± 4.34), especially if compared to the healthy subjects (mean = 0.37 ± 0.32). This is consistent with what the authors would usually expect from patients’ reports. Overall, these results demonstrate that the Italian version of the svQOD-NS is a reliable and effective tool, and useful in the context of a personalized approach for the assessment of olfactory dysfunction.

This study is subject to limitations. According to previous research, in humans, olfactory sense is associated not only with QoL, but also with individual safety [34]. Therefore, decline in sense of pleasure due to OD could lead to neglect in many daily-life activities, raising safety concerns [35]. In the ITA svQOD-NS, the two items (#4, #5) concerning food-related behavior due to OD may as a result be skewed towards the sense of pleasure rather than safety. This inaccuracy, however, is secondary to the inclusion of the seven items, as already validated in the other versions of the svQOD-NS by other esteemed authors [24,25,26]. Secondly, the validation of this questionnaire was performed in a group of patients suffering from either CRSwNP or SD, thus its external validity was not assessed. Further analyses with other cohorts of patients will be helpful in verifying the applicability of the ITA svQOD-NS in cases of OD with different etiologies. Finally, the authors believe that the improvement of the ITA svQOD-NS through the addition of fear-conditioning statements, aimed to assess the role of OD also on individual safety, will be the basis for future publications.

## 5. Conclusions

In this study, we assessed the validity of the Italian version of the svQOD-NS, which showed reliable consistency, test-retest reliability, and significant correlation with all of the most used clinical questionnaires. The ITA svQOD-NS can be efficiently adopted to evaluate the impact of OD on QoL in the Italian population in everyday clinical practice.

## Figures and Tables

**Table 1:** Characteristics of the population. sd = standard deviation; NPS = Nasal Polyps Score.

		Cases	Controls
Gender	females (%)	21 (42)	26 (52)
	males (%)	29 (58)	24 (48)
Age	mean ± sd	44.1 ± 13.9	43.0 ± 15.1
Disease	CRSwNP (%)	25 (50)	/
	septal deviation (%)	25 (50)	/
NPS	mean ± sd	4.7 ± 2.3	/
Comorbidities	asthma (%)	13 (26)	4 (8)
	allergy (%)	22 (44)	6 (12)
Smoking	yes (%)	10 (20)	12 (24)
	no (%)	40 (80)	38 (76)

**Table 2:** Overall scores from each questionnaire among cases pre-operatively (T0), one month (T1) and nine months after surgery (T2). sd = standard deviation.

	Timepoint		
Questionnaire	T0 (±sd)	T1 (±sd)	T2 (±sd)
ITA svQOD-NS	8.58 ± 5.70	2.33 ± 3.11	1.49 ± 1.72
SNOT-22	46.46 ± 19.11	19.42 ± 12.63	10.88 ± 8.12
VAS	6.10 ± 3.10	2.02 ± 2.56	0.98 ± 1.12
HADS	9.32 ± 6.92	6.02 ± 6.29	3.86 ± 3.31

**Table 3:** Internal consistency of the ITA svQOD-NS; item-to-total correlations and Cronbach’s α if item is deleted.

ITEM of the ITA svQOD-NS	Item-to-Total Correlation	Cronbach’s α if Item Deleted
item#1	0.646	0.922
item#2	0.780	0.909
item#3	0.789	0.908
item#4	0.767	0.911
item#5	0.744	0.913
item#6	0.850	0.902
item#7	0.757	0.913

**Table 4:** Mean scores for each item (range: 0–3) and total score (range: 0–21) of the ITA svQOD-NS. In each cell, mean value ± standard deviation are reported.

ITA svQOD-NS	Cases + Controls	CRSwNP	Septal Deviation	Healthy Controls
item#1	0.96 ± 1.05	1.45 ± 1.06	0.43 ± 0.34	0.04 ± 0.03
item#2	1.61 ± 1.11	2.18 ± 0.80	0.97 ± 0.76	0.06 ± 0.04
item#3	1.43 ± 1.17	2.00 ± 0.99	0.83 ± 0.73	0.08 ± 0.05
item#4	0.70 ± 1.00	1.18 ± 1.14	0.19 ± 0.14	0.02 ± 0.02
item#5	0.86 ± 1.08	1.34 ± 1.10	0.35 ± 0.29	0.02 ± 0.01
item#6	1.39 ± 1.16	1.92 ± 1.00	0.81 ± 0.67	0.06 ± 0.04
item#7	1.54 ± 1.13	2.16 ± 0.92	0.95 ± 0.81	0.08 ± 0.05
TOTAL	4.47 ± 5.80	12.23 ± 5.31	4.53 ± 4.34	0.37 ± 0.32

**Table 5:** Differences of mean total scores of the ITA svQOD-NS in the various subgroups. SD = septal deviation; CI = confidence interval.

ITA svQOD-NS		Score Difference	95%CI	*p* Value
in CRSwNP	vs. SD	+7.7	3.82–8.75	<0.001
	vs. Controls	+11.86	9.26–13.62	<0.001
in SD	vs. Controls	+4.16	3.06–7.34	<0.001

**Table 6:** Difference of the means of each score at different time points: pre-operatively (T0), one month (T1) and nine months after surgery (T2). sd = standard deviation; CI = confidence interval.

Questionnaire	Difference of the Means (±sd)	95%CI	*p* Value
ITA svQOD-NS			
T0 vs. T1	6.14 ± 5.28	4.64–7.64	<0.001
T1 vs. T2	0.84 ± 1.41	0.44–1.24	<0.001
T0 vs. T2	6.98 ± 5.27	5.48–8.48	<0.001
SNOT-22			
T0 vs. T1	27.04 ± 17.90	21.95–32.12	<0.001
T1 vs. T2	8.54 ± 7.73	6.34–10.74	<0.001
T0 vs. T2	35.58 ± 18.58	30.30–40.86	<0.001
VAS			
T0 vs. T1	4.08 ± 3.15	3.19–4.97	<0.001
T1 vs. T2	1.04 ± 1.47	0.62–1.46	<0.001
T0 vs. T2	5.12 ± 2.90	4.29–5.95	<0.001
HADS			
T0 vs. T1	3.30 ± 5.40	1.77–4.83	<0.001
T1 vs. T2	2.16 ± 2.35	1.49–2.83	<0.001
T0 vs. T2	5.46 ± 5.20	3.98–6.94	<0.001

## Data Availability

Not applicable.
